# G Protein-Coupled Receptor 109A and Host Microbiota Modulate Intestinal Epithelial Integrity During Sepsis

**DOI:** 10.3389/fimmu.2018.02079

**Published:** 2018-09-13

**Authors:** Guangxin Chen, Bingxu Huang, Shoupeng Fu, Bai Li, Xin Ran, Dewei He, Liqiang Jiang, Yuhang Li, Bingdong Liu, Liwei Xie, Juxiong Liu, Wei Wang

**Affiliations:** ^1^College of Veterinary Medicine, Jilin University, Changchun, China; ^2^Institutes of Biomedical Sciences, Shanxi University, Taiyuan, China; ^3^First Hospital of Jilin University, Changchun, China; ^4^State Key Laboratory of Applied Microbiology Southern China, Guangdong Provincial Key Laboratory of Microbial Culture Collection and Application, Guangdong Open Laboratory of Applied Microbiology, Guangdong Institute of Microbiology, Guangzhou, China; ^5^First Affiliated Hospital of Jinan University, Guangzhou, China

**Keywords:** GPR109A, gut microbiota, intestinal epithelial barrier, CLP, sepsis

## Abstract

The intestinal epithelial barrier is important to mucosal immunity, although how it is maintained after damage is unclear. Here, we show that G protein-coupled receptor 109A (GPR109A) supports barrier integrity and decreases mortality in a mouse cecum ligation and puncture (CLP) sepsis model. Data from 16S RNA sequencing showed that the intestinal microbiota of *WT* and *Gpr109a*^−/−^ mice clustered differently and their compositions were disrupted after CLP surgery. GPR109A-deficient mice showed increased mortality, intestinal permeability, altered inflammation, and lower tight junction gene expression. After eliminating the intestinal flora with antibiotics, all experimental mice died within 48 h of CLP surgery. This demonstrates the critical role of the gut microbiota in CLP-induced sepsis. Importantly, mortality and other pathologies in the model were decreased after *Gpr109a*^−/−^ mice received *WT* gut microbiota. These findings indicate that GPR109A regulates the gut microbiota, contributing to intestinal epithelial barrier integrity and decreased mortality in CLP-induced sepsis.

## Introduction

Sepsis is a frequent cause of mortality in intensive care units and is one of the 10 most common causes of death worldwide (1–3). However, sepsis is not a single disease but a collection of several related syndromes that often occur after bacterial, viral, or fungal infection (4). Sepsis can also arise after sterile tissue injuries, such as pancreatitis, ischemia reperfusion injury, cancer, or other disorders (1). The intestine has been hypothesized to play a central role in the pathogenesis of sepsis and been referred to as the “motor” of the systemic inflammatory response (5–7). Sepsis is also often associated with impairment of the intestinal epithelial barrier. This increases intestinal permeability, causing the contents of intestinal lumen, including the microbiota, to leak out of the intestine, resulting in multiple organ failure or even death in the most extreme cases (8–10). The protective effects of the intestinal epithelial barrier are therefore essential for the prevention and treatment of sepsis.

In 2003, G protein-coupled receptor 109A (GPR109A) was identified as a receptor for niacin or nicotinic acid (NA) and was found to mediate the antilipolytic effects of NA (11–13). GPR109A belongs to the large G protein-coupled receptor family and is activated by 3-hydroxy-butyrate. Except for lymphocytes, it is expressed on most cells, including adipocytes, monocytes, macrophages, and dermal dendritic cells (14). GPR109A activation has been shown to reduce inflammation during atherosclerosis, sepsis, obesity, diabetic retinopathy, and renal disease (15–19). In recent years, GPR109A was thought to be associated with regulation of the microbiotametabolites, suppressing colonic inflammation, and maintaining intestinal barrier integrity (20, 21). However, dietary fiber has also been shown to regulate the composition of the intestinal microbiota (22–24). As the receptor for butyrate, previous studies have shown that microbiota-derived butyrate and NA inhibit inflammation of the intestinal tract by activating GPR109A in intestinal epithelial cells, dendritic cells, and macrophages (25). However, whether these microbiota-associated metabolites are the only associations that exist between the GPR109A receptor and the gut microbiota is unknown, as is the exact role that GPR109A plays in regulating the gut microbiota.

To investigate the function of GPR109A in more detail, our study used cecum ligation and puncture surgery (CLP), described as the “gold standard” model for sepsis(26), to examine the effects of *Gpr109a* deletion. We performed CLP surgery using *WT* and *Gpr109a*^−/−^ mice with a C57BL/6 background to investigate the protective effects of GPR109A receptor in this sepsis model. We also examined the association between GPR109A and the gut microbiota, in particular the role that the microbiota may play in controlling the pathology of sepsis. These experiments revealed that GPR109A regulates the intestinal microbiota, leading to the preservation of intestinal epithelial barrier integrity. This demonstrates the value of investigating the links between host and microbiota and may lead to improvements in sepsis therapy and control.

## Materials and methods

### Animals

*Gpr109a*^−/−^and *WT* mice were on a C57BL/6 background and maintained under specific pathogen-free (SPF) conditions. All mice were provided with food and water *ad libitum* and housed under a strict 12 h light-dark cycle. Six-to-eight week old female mice were used for all experiments. The *Gpr109a*^−/−^ was generous gift form Dr. Martin Sager (Zentrale Einrichtung für Tierforschung und Tierschutzaufgabender Heinrich-Heine Universität Düsseldorf, Germany). The *WT* mice were provided by the Centre of Experimental Animalsof the Baiqiuen Medical College of Jilin University (Changchun, China). Studies were performed in accordance with guidelines established by the Jilin University Institutional Animal Care and Use Committee (Permit number: 20170401).

### CLP model

CLP surgerywas performed as descried previously(27). Briefly, prior to CLP surgery, autoclaved the scissors and forceps, and anesthetized the mice with pentobarbital sodium (45 mg/kg). About 1 cm incision is made to the left ventral surface of the abdomen, and the cecum is exposed. The cecum is partially ligated (about 1 cm) at its base with a 3-0 silk suture, then the ligated cecum was punctured with a 21-gauge needle. The cecum was returned to the peritoneal cavity and the incision was sutured with 3–0 silk suture. The mice would recover in about 1 h.

### Body weight and temperature

Before the CLP surgery, measured the body weight using an electronic scale and recorded W-0, examined the rectal temperature with a thermometer and recorded T-0, after CLP surgery, measured the body weight and temperature at 6, 12, 24 and 48 h, and recorded W-6, W-12, W-24, W-48, and T-0, T-12, T-24, T-48 respectively. Weight loss-n = W-0 - W-n, Temperature change-n = T-0–T-n.

### Clinical score

During the experiment, roachback or emaciation, colon thickening and pellet morphology were recorded. The clinical score was calculated based on the scoring system as shown in Table 1.

**Table 1:** The scoring system of clinical score.

	**Roachback or Emaciation**	**Colon thickening**	**Pellet morphology**
0	None	None	Normal
1	Yes	Slight	Soft stool
2		Moderate	Diarrhea
3		Severe	Bloody stool

### Antibiotic treatment

Mice were given a combination of vancomycin (1 g/L), kanamycin (1 g/L), ampicillin (1 g/L), and metronidazole (1 g/L) in their drinking water following a previously published regimen (28, 29). After two months, colon samples were collected and incubated in lysogeny broth (LB) solid medium for 12 h to assess whether the gut microbiota had been completely removed by the antibiotics. All antibiotics used in this experiment were purchased from Sigma-Aldrich, St. Louis, MO, USA.

### Intestinal permeability assay

Intestinal permeability was assessed using fluorescein isothiocyanate (FITC)-dextran with an average molecular weight of 3,000–5,000 (Sigma-Aldrich), as previously described (30). Briefly, 2 d after CLP surgery, mice were deprived of food for 4 h and then gavaged with FITC-dextran (0.6 mg/g body weight at a concentration of 125 mg/mL). Four hours later, blood was collected from the eyes of micecentrifuged at 12,000 g for 3 min. The FITC-dextran content of the serum was determined using a microplate reader with an excitation wavelength of 490 nm and emission detection at 525 nm. Each sample was measured in triplicate.

### Fecal microbial transplantation

Between three to four pellets were collected from adult female mice, re-suspended in 2 mL sterile PBS/glycerol (80:20 v:v), and homogenized. Next, 200 μL of the homogenized fecal samples were introduced by gavage with a flexible plastic tube into the stomachs of experimental mice (*WT* intestinal microbiota transplanted into *Gpr109a*^−/−^ mice, *Gpr109a*^−/−^ intestinal microbiota transplanted into *WT* mice). The fecal suspension was administered three times, 7 d apart. The recipient mice were analyzed 14 d after the third microbiota transfer, as described previously (31).

### Gut microbiota profiling

Fresh colon samples were collected and immediately frozen using liquid nitrogen. These were then sent to Beijing Allwegene Gene Technology Co., Ltd., Beijing, China. Bacterial DNA was extracted using a PowerSoil DNA Isolation Kit (MoBio Laboratories, Carlsbad, CA, USA), following the manufacturer's instructions. The purity and quality of the genomic DNA were checked using 0.8% agarose gels. Bacterial DNA was PCR amplified with barcoded universal bacterial primers targeting the V3 and V4 variable regions of the 16S rRNA gene. Three PCR products per sample were pooled to mitigate reaction-level PCR biases. PCR products were purified using a QIAquick Gel Extraction Kit (Qiagen, Hilden, Germany), quantified using Real Time PCR, and sequenced at Auwigene, Beijing, China. Deep sequencing was performed using the Miseq platform at Auwigene. After sequencing, image analysis, base calling, and error estimation were performed using Illumina Analysis Pipeline Version 2.6 (Illumina, San Diego, CA, USA). The raw data were first screened and sequences removed if they were shorter than 200 bp, had a low-quality score (≤ 20), contained ambiguous bases, or did not exactly match the primer sequences and barcode tags. The remaining high-quality reads were separated using sample-specific barcode sequences and trimmed with Illumina Analysis Pipeline Version 2.6 for analysis using QIIME. The sequences were clustered into operational taxonomic units (OTUs) at a similarity level of 97% to generate rarefaction curves and to calculate richness and diversity indices. The Ribosomal Database Project (RDP) Classifier tool (https://rdp.cme.msu.edu/classifier/) was used to classify all sequences into different taxonomic groups. To examine the similarities between different samples, clustering analyses and PCoA were performed based on the OTUs from each sample using R. The evolutionary distances between microbial communities from each sample were calculated using thetayc coefficients and represented as an unweighted pair group method with arithmetic mean (UPGMA) clustering tree that described the dissimilarity (1-similarity) between multiple samples. These data were used to generate a Newick-formatted tree file. To compare group memberships and the structures of the communities in different samples, heat maps were generated using the 20 most abundant OTUs and mothur's Bayesian classifier.

### Hematoxylin and eosin (H&E)-staining and immunofluorescence

After CLP surgery 48 h later, mice were sacrificed and the colonic and ileac segments 2 to 3 cm in length were excised, washed in phosphate buffered saline (PBS), fixed in 4% formaldehyde, embedded in paraffin, and sectioned (5-μm thick). A portion of the paraffin sections were stained with hematoxylin and eosin (H&E) and the rest was used for immunofluorescence using procedures detailed previously (32). Briefly, antigens were unmasked by boiling under pressure in sodium citrate buffer. Tissue sections were cooled to room temperature (about 20°C) naturally and then washed with PBS three times (5 min each wash). The sections were incubated with a blocking buffer (5% donkey serum in PBS) for 60 min against the species of the secondary antibody. Primary antibodies (Mucin 2 H-300, rabbit polycional lgG, Santa Cruz, USA) were incubated at overnight 4°C and then the sections were washed three times with PBS. Alexa Fluor® 488 and 594, donkey anti-rabbit lgG (H+L; Life, USA) were diluted 1:1000 and incubated for 1 h at RT and washed three times in PBS. Nuclei were counterstained using DAPI. The tissue sections were then photographed with Nikon Eclipse Ti-U microscope and Nikon Intensilight C-HGFI (Japan).

### Quantitative real-time PCR (qRT-PCR)

After CLP surgery 48 h later, mice were sacrificed. Fresh colon and ileum tissues were collected and frozen using liquid nitrogen. These were then stored in a −80 °C ultra-low temperature freezer until use. The samples were pulverized with a cell/tissue grinder and total RNA was extracted using TRIzol (Invitrogen, Carlsbad, CA, USA), according to the manufacturer's protocol. cDNA was then generated using a commercial RT-PCR Kit (TaKaRa, Kyoto, Japan) and amplified with a SYBR Green QuantiTect RT-PCR Kit (Roche, Basel, Switzerland) and the primers of *IL-6, IL-1*β, *Cldn1, Cldn2, Cldn3, Ocln, Zo-1, Zo2*, and β-actin to evaluate the mRNA levels of various genes. Each of the samples was analyzed in triplicate using procedures referenced in our previous work (33). The sequences of primers used in this investigation are shown below.

**Table:** 

**Gene**	**Forward primer**	**Reverse primer**
*IL-6*	5′-AGCCACTGCCTTCCCTAC-3′	5′-TTGCCATTGCACAACTCTT-3′
*IL-1β*	5′-TGTGATGTTCCCATTAGAC-3′	5′-AATACCACTTGTTGGCTTA-3′
*Cldn1*	5′-AGGTCTGGCGACATTAGTGG-3′	5′-CGTGGTGTTGGGTAAGAGGT-3′
*Cldn2*	5′-AGTGGCTGTAGTGGGTGGAG-3′	5′-AAAGGATGACTCCGGCTACC-3′
*Cldn3*	5′-GAGATGGGAGCTGGGTTGTA-3′	GTAGTCCTTGCGGTCGTAGG
*Zo-1*	5′-GACCTTGATTTGCATGACGA-3′	5′-AGGACCGTGTAATGGCAGAC-3′
*Zo-2*	5′-CAGTCCCTATGCCTGAGAGC-3′	5′-TTGGAACCGCATAGATGTCA-3′
*Ocln*	5′-ACACTTGCTTGGGACAGAGG-3′	5′-AAGGAAGCGATGAAGCAGAA-3′
*β-actin*	5′-GTCAGGTCATCACTATCGGCAAT-3′	5′-AGAGGTCTTTACGGATGTCAACGT-3′

### Statistics

Data are presented as means ± SEM. Data were analyzed using the statistical software package SPSS 12.0 (SPSS Inc., Chicago, IL, USA). Groups were compared by one-way analysis of variance (ANOVA) followed by the least significant difference test. ^*^P < 0.05 was considered significant, and ^**^P < 0.01 was considered markedly significant.

## Results

### GPR109A-deficient mice show severe inflammation and increased mortality in a murine CLP-induced sepsis model

**Figure 1:**
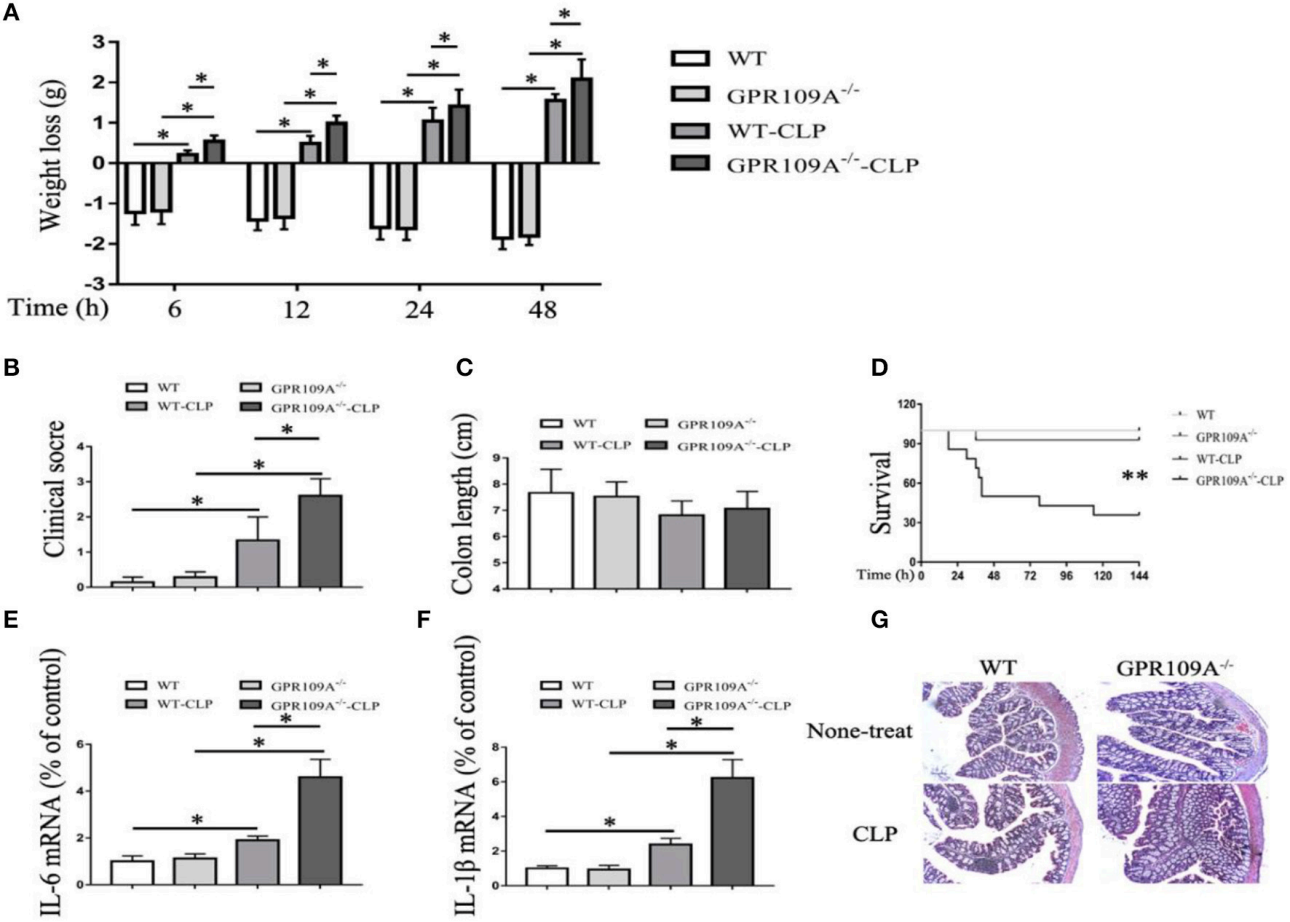
GPR109A-Deficient mice show an altered inflammatory response and increased mortality in a CLP-induced sepsis model. all measurements were performed using 6 to 8 week-old female mice, ^*^p < 0.05. **(A)** Body weight loss in none-treat *WT* and *Gpr109a*^−/−^mice, and CLP-treat *WT* and *Gpr109a*^−/^^−^ mice(*n* = 7 ~ 9), ^*^p < 0.05. **(B)** Clinical score of none-treat *WT* and *Gpr109a*^−/−^mice, and CLP-treat *WT* and *Gpr109a*^−/^^−^ mice (*n* = 5 ~ 9), ^*^p < 0.05. **(C)** The colon lengths of *WT* and *Gpr109a*^−/−^ mice, and CLP-treat *WT* and *Gpr109a*^−/−^ mice (*n* = 5 ~ 9). **(D)** The survival rates of none-treat *WT* and *Gpr109a*^−/−^, and CLP-treat mice (*n* = 14) mice, ^**^p < 0.01. **(E,F)** The expression levels of IL-6 and IL-1β mRNA in colon tissue homogenate from none- and CLP-treated *WT* and *Gpr109a*^−/−^ mice (*n* = 3, three determinations per mouse). The results shown are means ± SEM, ^*^p < 0.05. **(G)** Hematoxylin and eosin (H&E)-stained colon and ileum samples from none- and CLP-treated *WT* and *Gpr109a*^−/−^ mice (*n* = 3) mice. Magnification shown is 10×. The scale bar represents 500 μm.

Previous studies have reported that GPR109A inhibits inflammation during inflammatory bowel disease (IBD) (20, 21). To determine whether GPR109A functions in a similar manner during sepsis, we compared *WT* and *Gpr109a*^−/−^C57BL/6 female mice in a CLP-induced sepsis model. This showed that the pathogenic manifestations in *Gpr109a*^−/−^ mice were more serious than those in *WT* mice. These included more rapid loss of body weight, and more severe clinical scores, but there were no effects on colon length (Figures 1A–C). In particular, the *Gpr109a*^−/−^ mice had significantly decreased survival rate (Figure 1D), suggesting that GPR109A has a protective effect against CLP-induced sepsis in the model. These effects were also observed in colon tissue homogenate pro-inflammatory cytokine release (Figures 1E,F), indicating that GPR109A inhibited the expression of these pro-inflammatory markers during sepsis. We next examined the colon and ileum using hematoxylin and eosin (HE)-stained specimens (Figure 1G), revealing that GPR109A offered a protection against tissue damage during CLP-induced sepsis in the intestinal epithelium. All of these data indicate that GPR109A protects against intestinal inflammation in CLP-induced sepsis.

### GPR109A protects mice from CLP-induced intestinal permeability and a decrease in tight junction gene and mucin-2 protein expression

**Figure 2:**
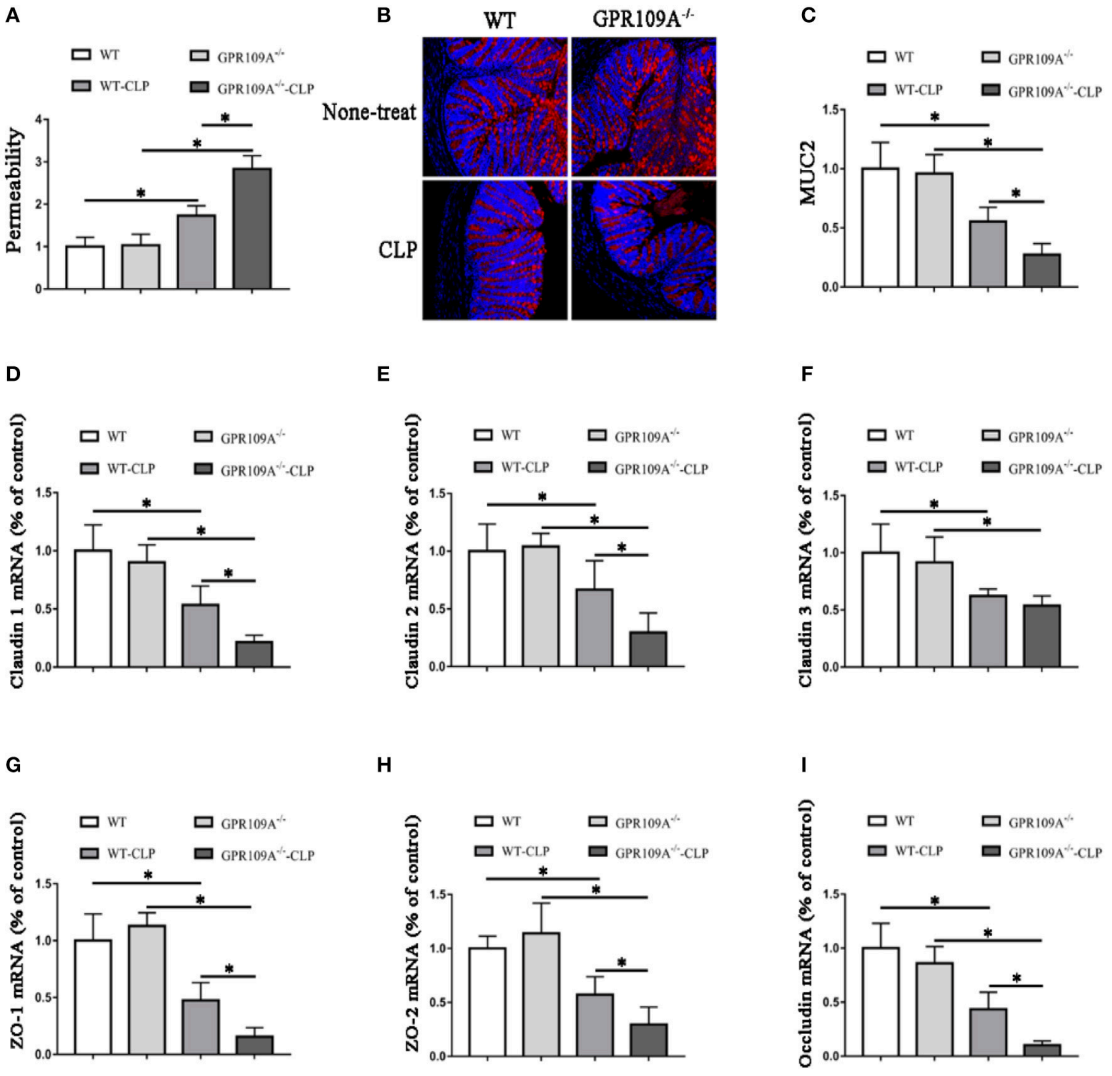
GPR109A Protects mice from CLP-induced intestinal permeability by regulating the expression of tight junction genes and Mucin-2 protein. **(A)** The intestinal of permeability of none- and CLP-treated *WT* and *Gpr109a*^−/−^ mice (*n* = 3, three determinations per mouse). The results shown are means ± SEM, ^*^p < 0.05. **(B)** Sections were prepared from the colons of none- and CLP-treated *WT* and *Gpr109a*^−/−^ mice (*n* = 3), and observed by fluorescence microscopy. Nuclear staining with DAPI is shown in blue. MUC2 staining is indicated in red. Magnification shown is 10×. The scale bar represents 500 μm. **(C)** Representative thequantization of MUC2 in B. **(D–I)** Representative the expression levels of claudin-1 (*Cldn1*), claudin-2 (*Cldn2*), claudin-3 (*Cldn3*), Zo1, Zo2, and occludin of colon tissue homogenate from none- and CLP-treated *WT* and *Gpr109a*^−/−^ mice (*n* = 3, three determinations per mouse). The results shown are means ± SEM, ^*^p < 0.05.

Although previous studies have shown that GPR109A protects against intestinal inflammation in IBD (20, 21), there was no investigation of the functions of GPR109A in maintaining intestinal barrier integrity in sepsis. To study intestinal leakage in *WT* and *Gpr109a*^−/−^ mice after CLP surgery, intestinal permeability was tested by orally administrating fluorescently-labeled FITC-dextran to the experimental mice. This showed decreased intestinal permeability in *Gpr109a*^−/−^ mice relative to that in *WT*, suggesting that GPR109A is involved in regulating permeability (Figure 2A). To further explore the mechanisms that underlie the effects of GPR109A on decreasing intestinal permeability, the expression of various tight junction gene transcripts in the colon was evaluated. This showed that the expression of claudin-1 (*Cldn1*), claudin-2 (*Cldn2*), *Zo1, Zo2*, and occludin (*Ocln*) was lower in *Gpr109a*^−/−^ mice relative to *WT* mice, although there was no difference in claudin-3 (*Cldn3*) expression (Figures 2D–I). We next examined the expression of mucin-2 (MUC2) protein using immunofluorescence. MUC2 is one of the main components of the mucosa and is typically expressed by goblet cells. We found that *Gpr109a*^−/−^ mice had less MUC2 protein expression than *WT*, indicating that GPR109A is involved in maintaining natural levels of MUC2 in *WT* mice (Figures 2B,C). We also measured the expression of tight junction genes and MUC2 in the ileum, demonstrating that these were consistent with the colon data (Figures S1A–G). All of these results indicate that GPR109A maintains the expression of tight junction genes and MUC2 protein, and has a role in controlling the permeability of the intestine in CLP-induced sepsis in mice.

### GPR109A regulates the gut microbiota and CLP-induced sepsis affects the composition of the intestinal microbiota

**Figure 3:**
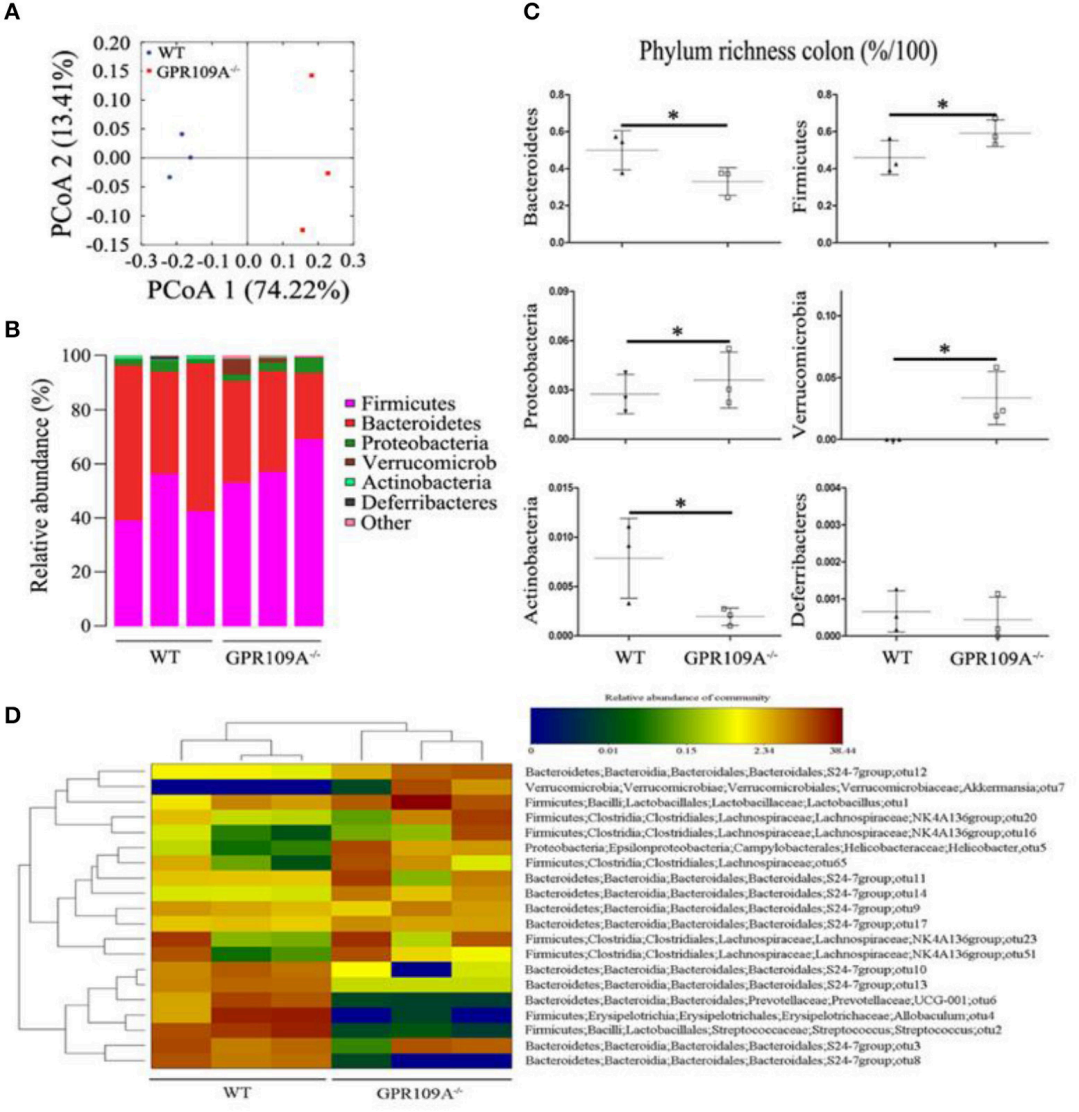
GPR109A regulates the gut microbiota of mice. **(A)** Principal coordinates analysis (PCoA) based on unweighted UniFrac distances of OTUs. Each symbol represents a single *WT* or *Gpr109a*^−/−^ colon sample (*n* = 3 for both). **(B)** Comparison of phylum-level proportional OTU abundances in *WT* or *Gpr109a*^−/−^ colon samples (*n* = 3 for both). **(C)** Representative proportions of OTUs classified at the phylum level (*n* = 3 mice for each). ^*^p < 0.05. **(D)** Heatmap depicting the OTU abundances at the phylum level within the *Bacteroidetes, Firmicutes, Proteobacteria*, and *Verrucomicrobia*. OTUs are shown as Phylum, Class, Order, Family, Genus, and Species.

Bacteria play a critical role in the pathogenesis of IBD and changes to the composition and abundances of the species in the gut microbiota can accompany the disease (34, 35). However, there are no reports on whether there is any alteration of the gut microbiota during sepsis. To investigate this, we performed 16S rRNA sequencing to assess the composition of the microbiota in colon samples from both *WT* and *Gpr109a*^−/−^ mice. A principal coordinates analysis (PCoA) using unweighted UniFrac distances showed that there were major differences between the gut microbiota of the two groups of mice (Figure 3A). As expected, the Firmicutes and Bacteroidetes were the most abundant phyla in samples (Figure 3B). However, the proportion that were Bacteroidetes or Actinobacteria was higher in the colons of *WT* mice compared to that in *Gpr109a*^−/−^ mice. Conversely, the Firmicutes, Proteobacteria, and Verrucomicrobia showed an opposite effect and were more abundant in the *Gpr109a*^−/−^ mice. There was no difference in the proportion that were Deferribacteres between both groups (Figure 3C). We next assessed if there was a difference in the abundances of operational taxonomic units (OTUs) at the phylum level in the Bacteroidetes, Firmicutes, Proteobacteria, and Verrucomicrobia. This analysis revealed that the OTUs present within the *WT*or*Gpr109a*^−/−^ mice clustered together, but the OTUs were different between*WT* and *Gpr109a*^−/−^mice (Figure 3D). We also compared the heterozygous Gpr109a^+/–^ and Gpr109a^–/–^ mice, and the results showed that there is obvious difference between the mice (Figure S2A). The Shannon, Simpson, PCoA and gut microbiota gene function and pathway analysis results showed that there was obvious difference on the intestinal microbiota composition of the Gpr109a^–/–^ and Gpr109a^+/–^, WT mice. The intestinal microbiota structure of Gpr109a^+/–^ is more similar than Gpr109a^–/–^ with WT mice (Figures S2B–E). All the results demonstrate that GPR109A regulates the structure of the gut microbiota in mice.

In order to understand the effects of CLP and sepsis on the gut microbiota, fecal samples were collected two days after surgery. Further 16S rRNA gene sequencing was used to analyze the microbiota and a PCoA showed that the individual differences in the sepsis mice were larger than in untreated mice (Figures S3A). This indicates that the compositions of gut microbiota were dramatically altered after CLP surgery. Notably, the proportions of Bacteroidetes increased by 25.87 and 56.57% in *WT* and *Gpr109a*^−/−^ mice compared to that in untreated mice, respectively. However, the proportions that were Firmicutes was reduced by 36.68 and 53.60% in *WT* and *Gpr109a*^−/−^ mice, respectively (Figures S3B,C). Changes to other phyla were also observed (Figure S2C). Compared to that in the untreated mice, OTU composition at the phylum level in the Bacteroidetes, Firmicutes, Proteobacteria, and Verrucomicrobia was also dramatically changed. Additionally, OTUs could not be clustered within the *WT* and *Gpr109a*^−/−^-CLP mice as before (Figure S3D). These data indicate that CLP-induced sepsis affects the structure of the gut microbiota in the mice colon.

### The gut microbiota protects against the effects of CLP-induced sepsis in the mouse model

**Figure 4:**
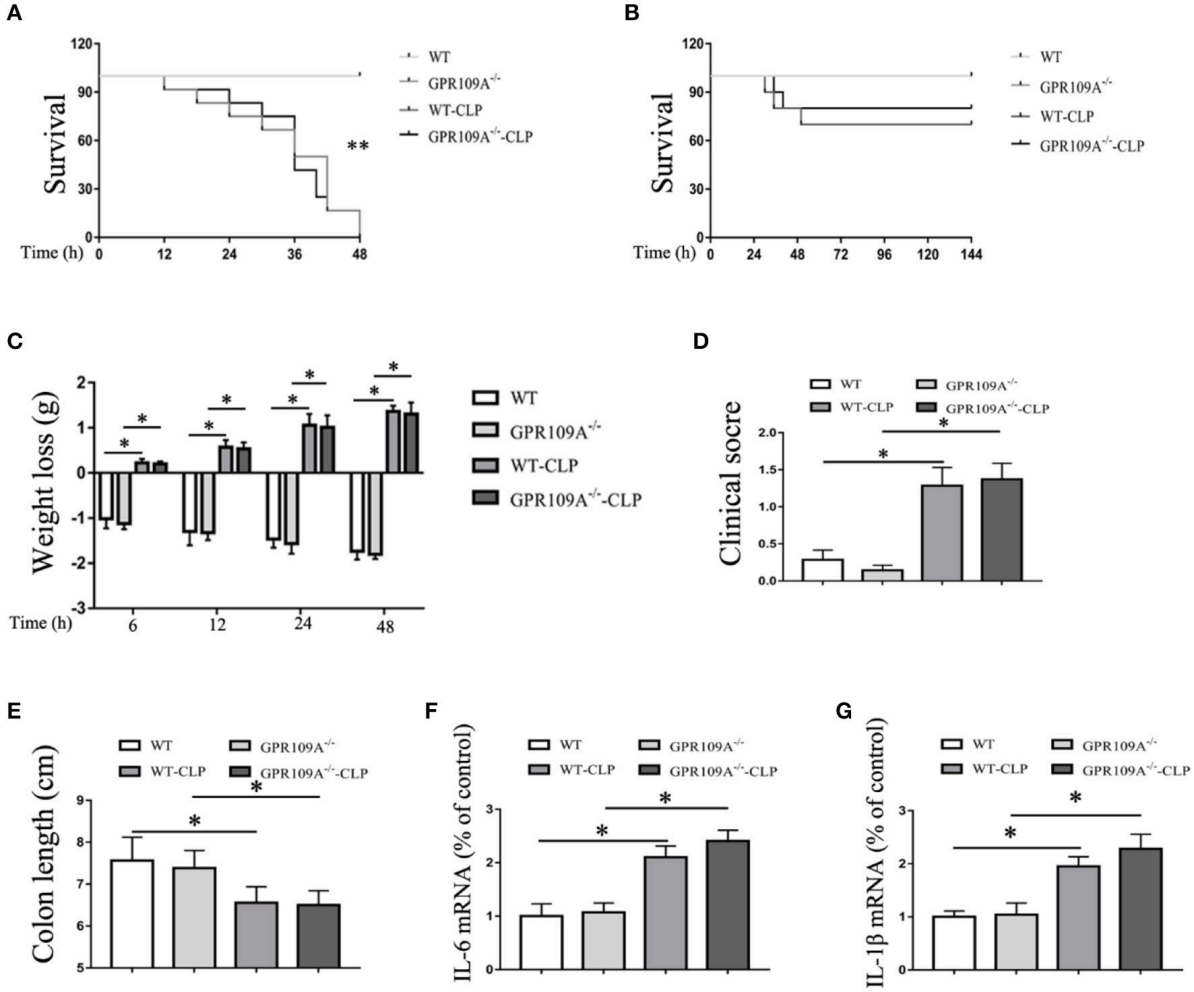
The gut microbiota plays a protective role during CLP-induced sepsis in mice. **(A)** The survival of none-treat *WT* and *Gpr109a*^−/–^ mice, andantibiotic treated *WT*and *Gpr109a*^−/–^ mice which undergo the CLP surgery (*n* ≥ 30)), ^**^p < 0.01. **(B)** Overall survival rate of none-treat *WT* and *Gpr109a*^−/–^ mice, and microbiota-transferred *WT* and *Gpr109a*^−/–^ mice which undergo the CLP surgery (*n* = 14). **(C)** Weight loss of none- and CLP-treated *WT* and *Gpr109a*^−/−^ mice (*n* = 7 ~ 9). **(D)** Clinical score of none- and CLP-treated *WT* and *Gpr109a*^−/−^ mice (*n* = 7 ~ 9), ^*^p < 0.05. **(E)** Colon lengths of different treatment mice (*n* = 7 ~ 9). **(F,G)** The expression of IL-6 and IL-1β mRNA in colon tissue homogenate from different treatment mice (*n* = 3).

In order to assess the role of the gut microbiota during CLP-induced sepsis, we used orally administered antibiotics and gut flora transplantation to either remove or replace the microbiota. *WT* and *Gpr109a*^−/−^ mice were administered a combination of vancomycin (1 g/L), kanamycin (1 g/L), ampicillin (1 g/L), and metronidazole (1 g/L) in their drinking water for two months using a validated regimen (28, 29) (Figure S4A). Colon samples were collected and incubated in lysogeny broth (LB) solid medium for 12 h. Intact colonies were observed in the *WT*-Sham (W-Sham) and *Gpr109a*^−/−^-Sham (G-Sham) groups. However, the antibiotic treated groups displayed negligible colony growth (Figure S4B), demonstrating that the gut microbiota had been removed by the antibiotics. We then examined the effects of antibiotic treatment and CLP-induced sepsis. During this experiment, all of the mice died within 48 h (*n* ≥ 30) of CLP surgery (Figure 4A). This demonstrates that the gut microbiota plays a critical role in protection against CLP-induced sepsis in the mouse model.

**Figure 5:**
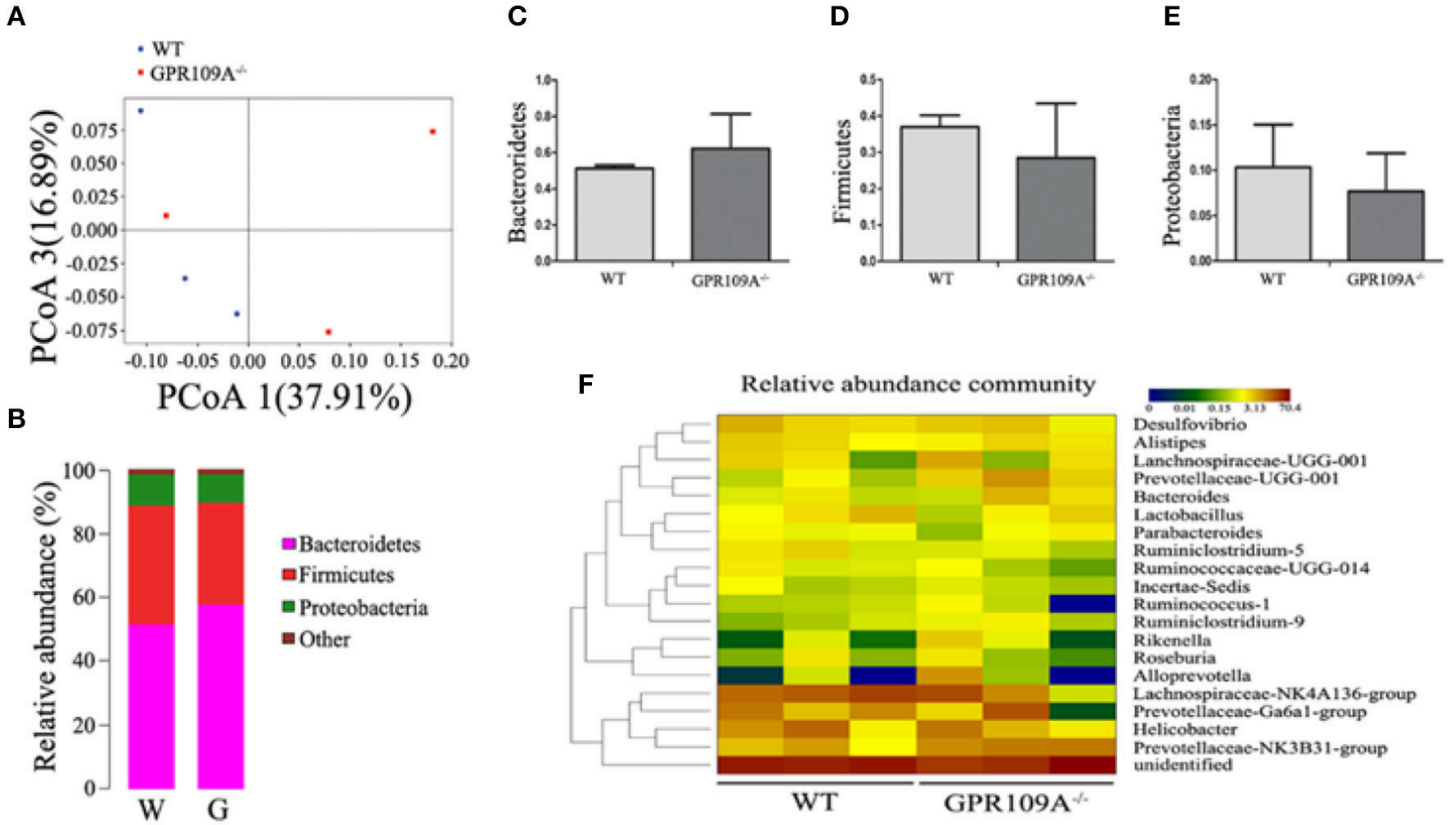
16S rRNA Sequencing of colon samples from microbiota transferred mice. **(A)** Principal coordinates analysis (PCoA) based on the unweighted UniFrac distances of OTUs. Each symbol represents a single colon sample from *WT* (*n* = 3) or *Gpr109a*^−/–^ (*n* = 3) mice. **(B)** Representative comparisons of the average phylum-level proportional abundances in colon samples from *WT* (*n* = 3) and *Gpr109a*^−/–^ (*n* = 3) mice. **(C–E)** Representative proportions of OTUs classified at the phylum level (*n* = 3 for each). **(F)** Heatmap depicting the top 20 genera in the gut microbiota of colon samples from *WT* (*n* = 3) and *Gpr109a*^−/–^ (*n* = 3) mice.

To validate this hypothesis, we transplanted the intestinal flora of *WT* mice into *Gpr109a*^−/−^ mice via fecal transfer. After sequencing the gut microbiota of the microbiota-transplanted mice, the PCoAresultsshowed large individual differences (Figure 5A), but the relative abundances of phyla were almostidentical in the Bactericides, Firmicutes, and Proteobacteria (Figures 5B–E). We then examined the 20 most abundant genera in the gut microbiota, revealing that there were no differences betweenthe *WT*mice (receive the intestinal microbiota of *Gpr109a*^−/−^ mice) and *Gpr109a*^−/−^mice (receive the intestinal of *WT* mice; Figure 5F). This indicated that the gut flora had been successfully transferred between the *WT* and *Gpr109a*^−/−^ mice. CLP surgery was then performed in these gut microbiota transplanted mice, revealing that there were no differences between *WT* and *Gpr109a*^−/−^ mice when assessed using various pathological measures, including lossof body weight, change in body temperature, clinical score, colon length, and overall survival (Figures 4B–E). The expression of colon epithelium pro-inflammatory cytokines was also similar between the *WT* and *Gpr109a*^−/−^mice (Figures 4F,G). The results demonstrated that there is no pathological differences between *WT* mice and *Gpr109a*^−/−^mice with a *WT* microbiota.

**Figure 6:**
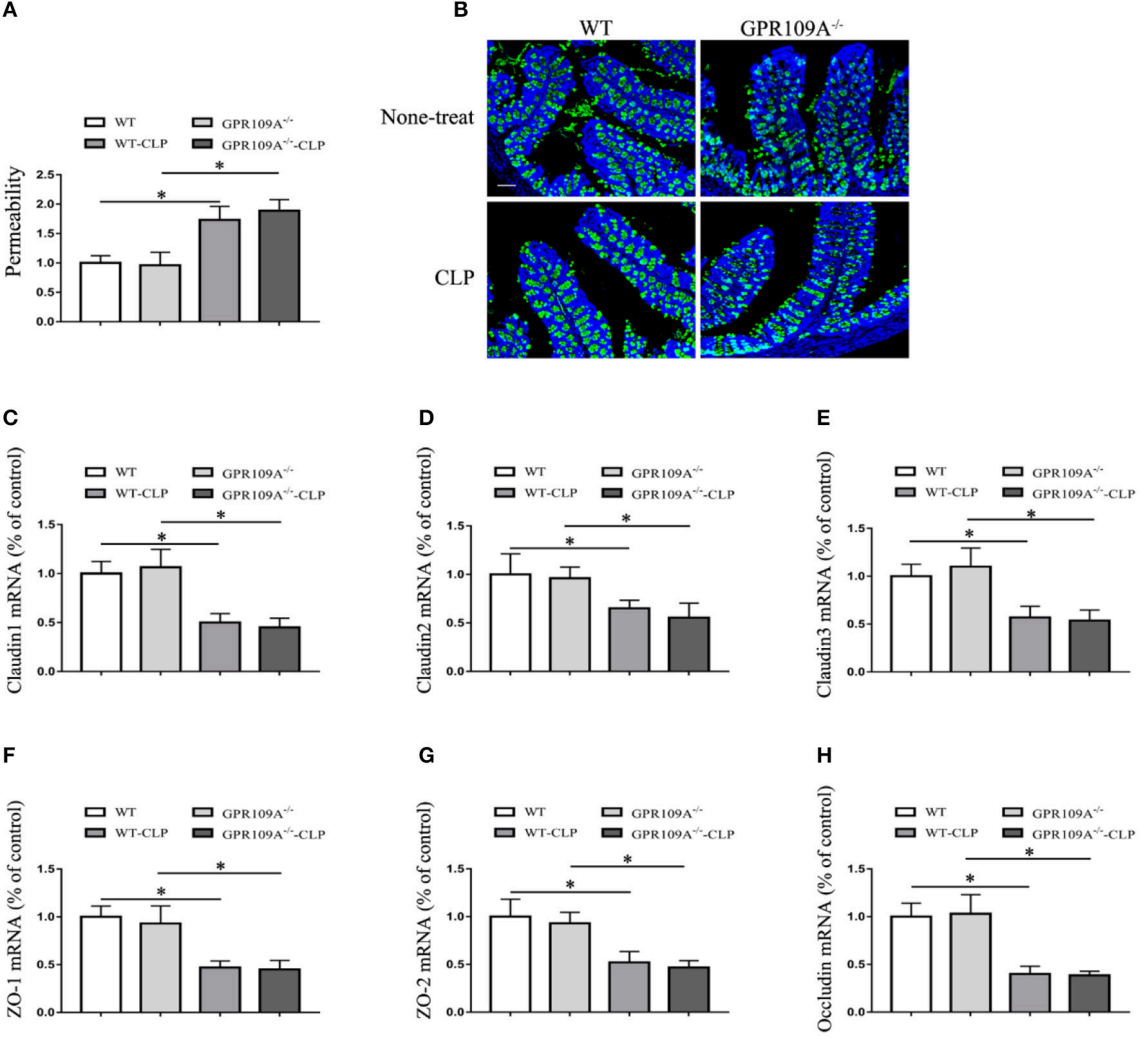
The gut microbiota affects intestinal permeability and promotes the natural expression of MUC2 protein and tight junction genes in the CLP-induced Sepsis Model. **(A)** The intestinal of permeability of different treatment mice (*n* = 3). The results are shown as means ± SEM, ^*^p < 0.05. **(B)** Tissue sections were prepared from the colons of none-treat *WT* and *Gpr109a*^−/–^ mice, and microbiota-transferred *WT* and *Gpr109a*^−/–^ mice which undergo the CLP surgery (*n* = 3) and observed by fluorescence microscopy. The nucleus was stained with DAPI (blue) and MUC2 was stained green. Magnification shown is 10×. The scale bar represents 500 μm. **(C–H)** Representative expression levels of claudin-1 (*cldn1*), claudin-2 (*cldn2*), claudin-3 (*cldn3*), *ZO-1, ZO-2*, and occludin (*ocln*) in colon tissue homogenates from different treatment mice (*n* = 3). The results shown are means ± SEM, ^*^p < 0.05.

To further investigate the involvement of host microbiota, we examined the permeability of microbiota-transferred mice. This showed that there was no difference between *WT* and *Gpr109a*^−/−^ mice after transplantations of intestinal microbiota (Figure 6A). We next measured MUC2 protein expression using immunofluorescence and the expression of tight junctions genes by qRT-PCR. This again showed that there were no differences between the two groups after transfer of a *WT* microbiota into *Gpr109a*^−/−^ mice and *Gpr109a*^−/−^microbiota into *WT* mice (Figures 6B–H). We also measured tight junction gene expression and MUC2 protein in ileum samples, demonstrating that they were similar to those in the colon samples (Figures S5A–G). Together, these results indicate that the gut microbiota has a protective effect against CLP-induced sepsis in the mouse model.

## Discussion

The intestinal epithelium is a critical barrier that protects against potentially harmful microbiota and pathogens (36). Defective tight junctions in the intestinal epithelial barrier can lead to an increase in intestinal permeability, contributing to the development of intestinal inflammation (37, 38, 39). After organisms and viruses from the microbiota cross the intestinal epithelium and enter the blood, they can result in multi-organ dysfunction and even death (8–10). The protective effects of the intestinal epithelium barrier are therefore central to the prevention and treatment of sepsis.GPR109A have been demonstrated plays an important role in maintaining intestinal epithelium barrierand inhibiting inflammationby us and others (21, 40).

In the present study, we have examined the differences in survival, inflammation, and epithelial barrier integrity in *WT* and *Gpr109a*^−/−^ mice using a CLP-induced model of sepsis. This has demonstrated that *Gpr109a*^−/−^mice showed overall lower survival, altered inflammation, and disruption of epithelial barrier integrity. This is consistent with previous studies that indicate that microbial-derived butyrate protects against inflammation through GPR109A signaling during IBD (20, 21). Butyrate is one of the most important microbiota metabolites involved in the activation of the GPR109A receptor. Activation by butyrate and NA in intestinal epithelial cells, dendritic cells, and macrophages, can lead to inhibition of inflammation in the intestinal tract (25). However, there have been no studies investigating the direct influence of host GPR109A on the intestinal microbiota, although recent studies have revealed that dietary fiber can help to maintain the stability of the intestinal flora (22–24).

To investigate the relationship between GPR109A and the microbiota, we performed 16S rRNA sequencing using colon fecal samples from *WT* and *Gpr109a*^−/−^ mice, demonstrating that the GPR109A receptor regulated the structure of the gut microbiota and that the microbial populations were different between *WT* and *Gpr109a*^−/−^mice. Bacteroidetes and Firmicutes, two major phyla of gut microbiota in humans, which are involved in the regulation of lipid and bile acid metabolism in the gut(41). Proteobacteria is phylum of Gram-negativebacteria includes many pathogenic and lipopolysaccharide-containing bacteria, such as *Escherichia*. Jacqueline M. reported a relative high abundance of Proteobacteria was observed in critically ill patients (42). In particular, the proportions of Firmicutes, Actinobacteria, Bacteroidetes, Proteobacteria, and Verrucomicrobia we observed differed between the two groups of mice (Figure 3C). It's remarkable that the abundance of Proteobacteria is higher in *Gpr109a*^−/−^mice than control. The OTU abundances at the phylum level within the Bacteroidetes, Firmicutes, Proteobacteria, and Verrucomicrobia were also different (Figure 3D). Similar research has revealed that mice deficient in the intestinal inflammatory regulator NLRP12 possess a dysbiotic microbiome and exacerbated colitis (43). Additionally, SPF mice have been found to display different inflammatory responses in the gut depending on the composition of their intestinal microbiota in a DSS-induced model of colitis (44). These data, combined with our results, indicate that that GPR109A and the microbiota interact to maintain homeostasis in the host epithelial barrier.

Our data also showed that the gut microbiota in the *WT* and *Gpr109a*^−/−^ groups changed dramatically after CLP-induced sepsis relative to that in untreated mice. This indicated that CLP-induced sepsis severely disrupted the composition of the gut microbiota, leading to dysbiosis. The ratio of Firmicutes to Bacteroidetes (F/B ratio) was thought as amicrobiota marker in critically ill patients which represent decreased microbial diversity and severe inflammatory response. Ojima M have reported that extreme changes in the F/B ratio were observed in almost all of the sepsis patients with a poor prognosis (45). In the present study, *Gpr109a*^−/−^mice showedextraordinary decrease in F/B ratio after CLP (data was not shown) and eventually lead to an increase in mortality.Previous work using TNF^deltaARE^ mice has also shown that inflammation associates with the development of dysbiosis (32). Several other diseases have also been shown to associate with imbalances in the composition of the gut microbiota, although whether dysbiosis contributes directly to the development of these diseases, or is a reflection of the altered physiological state of the host, is still debated (46–48). Bacteroidetes and Firmicutes are the predominant bacterial phyla colonizing the healthy human large intestine that both ferment dietary fiber, they are the main producer of butyrate which have been authenticated contribute to intestinal health. To investigate the potential role of the microbiota in the sepsis model, we examined the effects of CLP surgery in mice with their microbiota removed via antibiotics. Surprisingly, all the experimental mice died within 48 h, suggesting that the gut microbiota diversity is crucial for survival in the CLP-induced sepsis model. Accordinglymicrobiota deletion exacerbate the severity of CLP induced sepsis. To further investigate the role of the gut microbiota in the CLP-induced sepsis model, we transplanted the gut microbiota of *WT*and*Gpr109a*^−/−^into each other and performed CLP surgery. This revealed increased survival in the *Gpr109a*^−/−^ mice that received the *WT* microbiota compared to that in control mice, and there was no significant change after the *WT* mice received the microbiota from *Gpr109a*^−/−^ mice. There were also changes in the expression of inflammatory markers and improved epithelium barrier integrity in the CLP-induced sepsis model. This is supported by prior work demonstrating that the gut microbiota from wild mice can be banked and maintained in a genetically similar laboratory mouse colony. Transfer of the more natural gut microbiota from the wild mice improved outcomes during viral infection and in tumorigenesis models (49). Another study also showed attenuation of colonic inflammation in *Nlrp12*^−/−^ mice using fecal transplants (43).

In summary, our results indicate that the GPR109A receptor contributes to improved survival, altered inflammatory marker expression, and increased gut epithelium barrier integrity in a CLP-induced sepsis model. We have also demonstrated that GPR109A is involved in regulating the gut microbiota of mice, although the underlying mechanisms remain unclear. Despite this, our work demonstrates the important role that the microbiota and GPR109A play during sepsis and may lead to improved treatment and diagnosis for patients.

## Author contributions

GC, LX, JL, and WW designed the experiments. GC, BH, and SF performed the experiments and analyzed data. BL did the H&E staining. XR, DH, LJ, YL, and BdL participated in the CLP surgery. BdL from Xie Lab at Guangdong Institute of Microbiology carried out 16S rRNA sequencing analysis. GC, LX, and WW collected the data and wrote the manuscript.

### Conflict of interest statement

The authors declare that the research was conducted in the absence of any commercial or financial relationships that could be construed as a potential conflict of interest.
