# Supplementary material for: G Protein-Coupled Receptor 109A and Host Microbiota Modulate Intestinal Epithelial Integrity During Sepsis
Source: Front Immunol. 2018 Sep 13;9:2079. doi: 10.3389/fimmu.2018.02079 (PMC6146091; doi:10.3389/fimmu.2018.02079)
Supplement: Supplementary file 1 [file Data_Sheet_1.pdf]

Supplemental Figures and Legends

Figure S1. The Expression of Tight Junction Genes and MUC2 After CLP Surgery

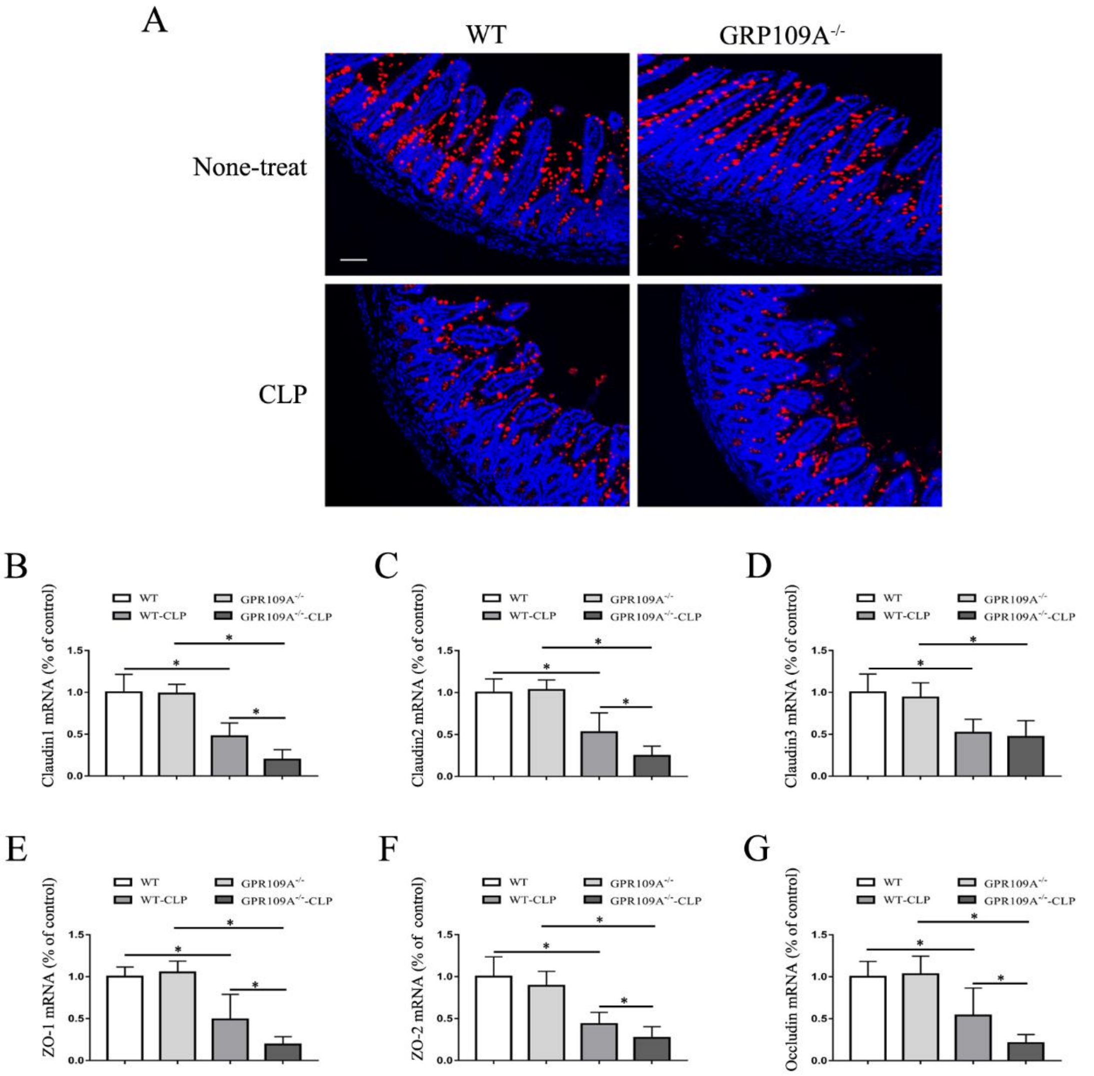

Figure S1. The Expression of Tight Junction Genes and MUC2 After CLP Surgery. (A) Sections were prepared from the ileum of *WT* (n = 3), *Gpr109a*$^{-/-}$ mice (n = 3) and CLP treated mice. The nucleus was stained with DAPI and is shown in blue. MUC2 staining is indicated in red. The sections were observed by fluorescence microscopy. Magnification shown is 10×. The scale bar represents 500 μm. (B-G) Representative expression levels of claudin-1 (*Cldn1*), claudin-2 (*Cldn2*), claudin-3 (*Cldn3*), *Zo-1*, *Zo-2*, and occludin (*Ocln*) in ileum tissue homogenates from different treatment mice (n = 3, three determinations per mouse). The results shown are means ± SEM, *p < 0.05.

Figure S2. GPR109A Regulates the Intestinal Microbiota of Mice

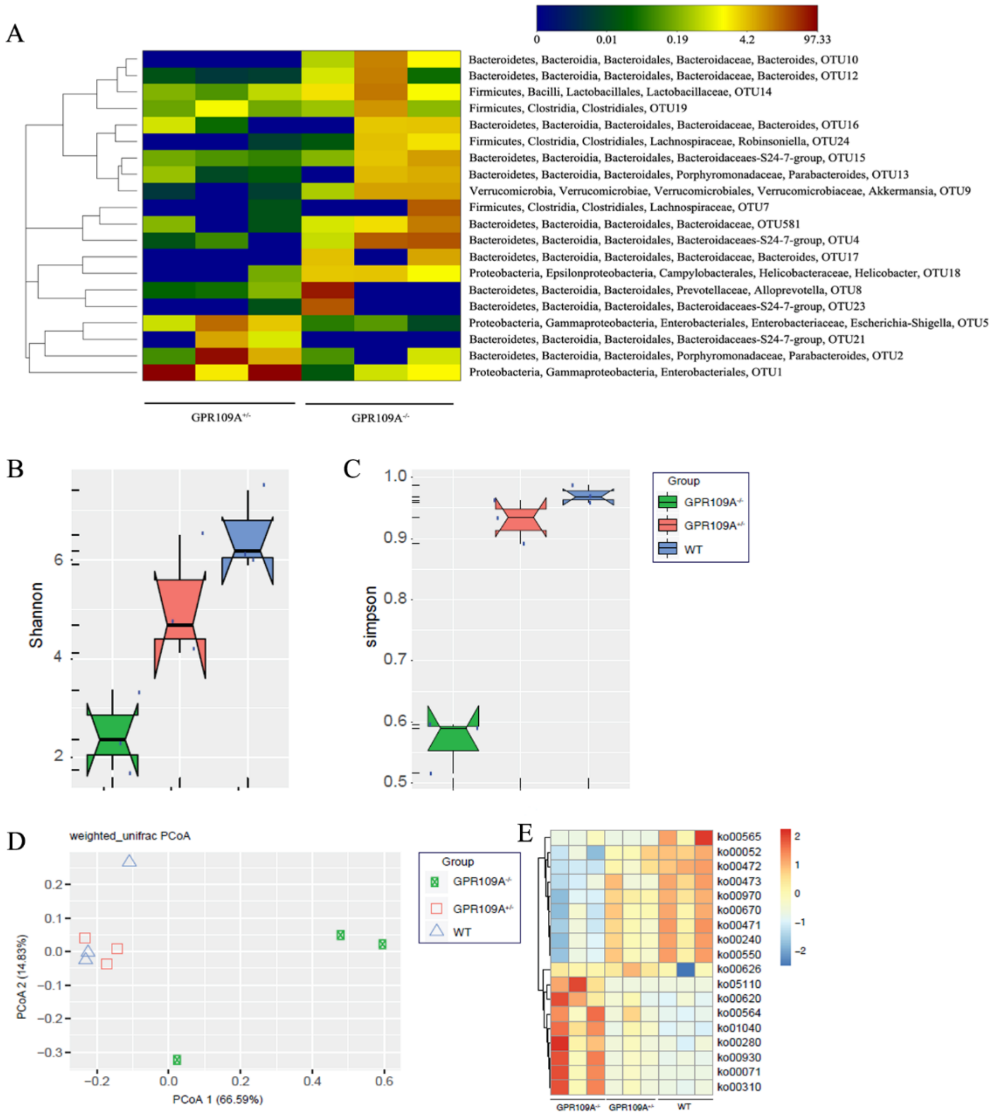

Figure S2. GPR109A Regulates the Intestinal Microbiota of Mice. (A) Heatmap depicting the abundances of operational taxonomic units (OTUs) at the phylum level within the Bacteroidetes, Firmicutes, Proteobacteria, Verrucomicrobia, and Tenericutes. OTUs are shown as Phylum, Class, Order, Family, Genus, and Species. (B, C) The Shannon and Simpson analysis of intestinal microbiota structure of *WT*, *Gpr109a*$^{-/-}$ and *Gpr109a*$^{+/-}$ mice (n = 3). (D) Principal coordinates analysis (PCoA) based on unweighted UniFrac distances of OTUs. Each symbol represents a single colon sample from *WT*, *Gpr109a*$^{-/-}$ and *Gpr109a*$^{+/-}$ (n = 3) mice. (E) The gut microbiota gene function and pathway analysis of *WT*, *Gpr109a*$^{-/-}$ and *Gpr109a*$^{+/-}$ mice (n = 3).

Figure S3. CLP-Induced Sepsis Disrupts the Gut Microbiota of Mice

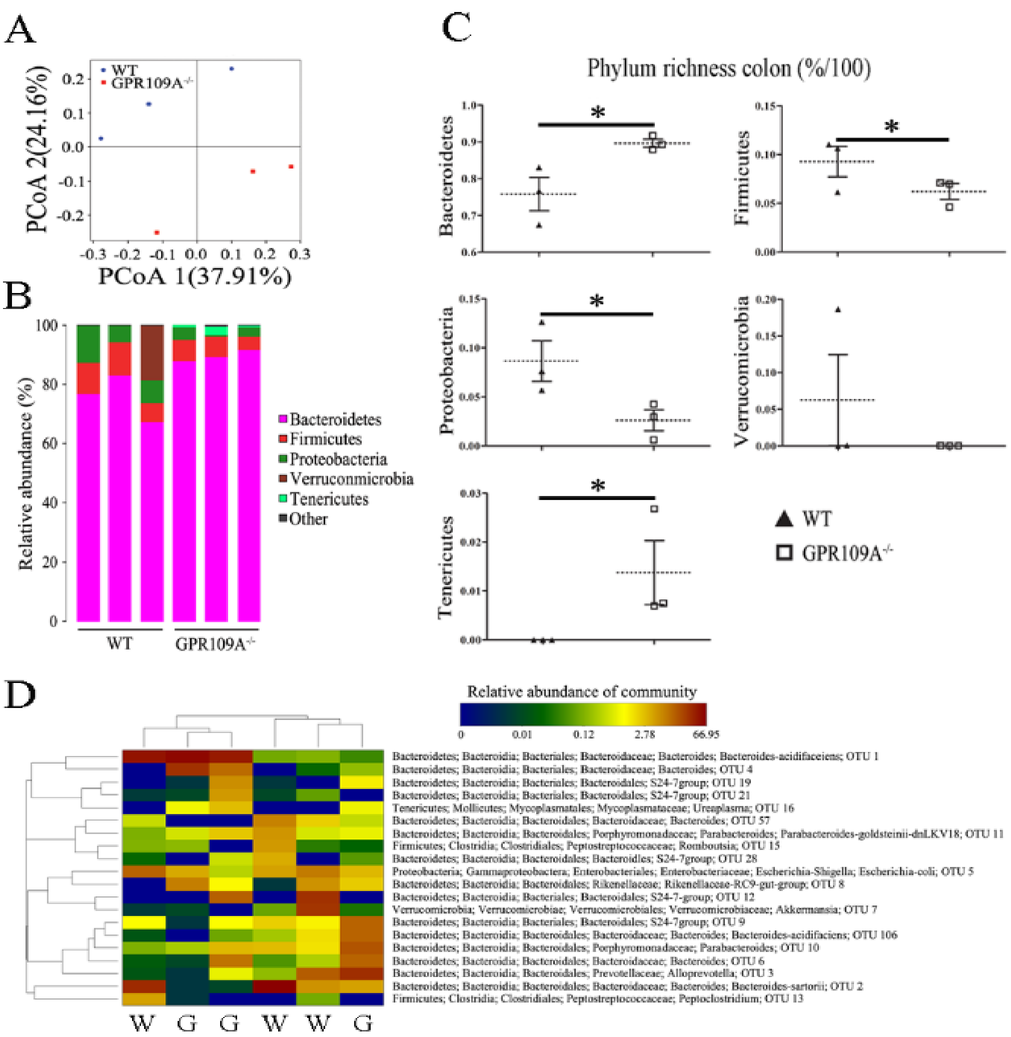

Figure S3. CLP-Induced Sepsis Disrupts the Gut Microbiota of Mice. (A) Principal coordinates analysis (PCoA) based on unweighted UniFrac distances of OTUs. Each symbol represents a single colon sample from *WT* (n = 3) and *Gpr109a*$^{-/-}$ (n = 3) mice. (B) A comparison of the phylum-level proportional abundances of colon samples from *WT* (n = 3) and *Gpr109a*$^{-/-}$ (n = 3) mice. (C) Representative proportions of OTUs classified at the phylum-level. Each contains data from three mice. *$p < 0.05$. (D) Heatmap depicting the abundances of operational taxonomic units (OTUs) at the phylum level within the Bacteroidetes, Firmicutes, Proteobacteria, Verrucomicrobia, and Tenericutes. OTUs are shown as Phylum, Class, Order, Family, Genus, and Species.

Figure S4. Antibiotic Treated *WT* and *Gpr109a*$^{-/-}$ mice

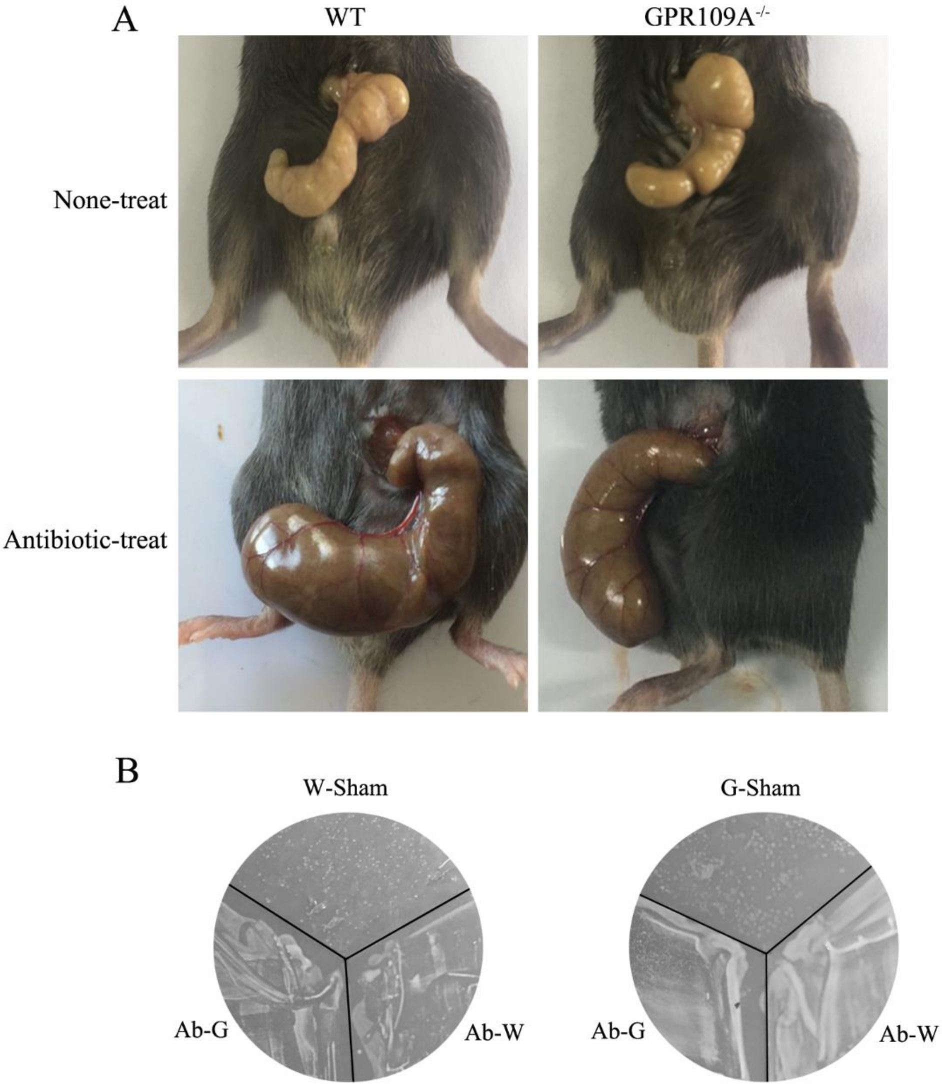

Figure S4. Antibiotic Treated *WT* and *Gpr109a*$^{-/-}$ mice. (A) Images of representative *WT* and *Gpr109a*$^{-/-}$, and mice were treated with antibiotics for day 60. (B) After antibiotic treatment for two months, colon faeces samples were collected and incubated in lysogeny broth (LB) solid medium for 12 h. Ab-G represents antibiotic-treated *Gpr109a*$^{-/-}$ mice while Ab-W represents antibiotic treated *WT* mice. W-Sham are control *WT* mice and G-Sham are control *Gpr109a*$^{-/-}$ mice.

Figure S5. The Expression of Tight Junction Genes and MUC2 Protein After CLP Surgery in Gut Microbiota-Transferred Mice

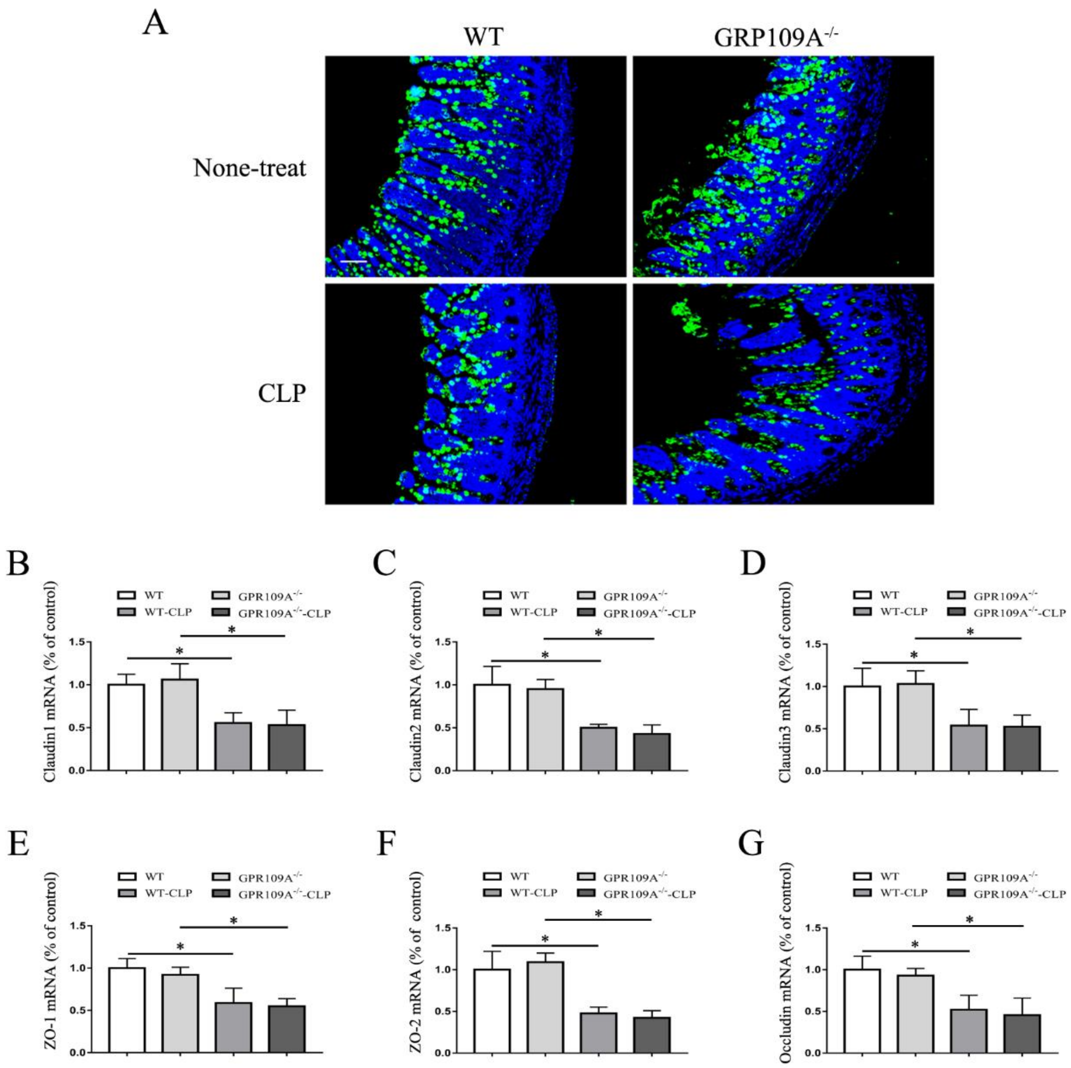

Figure S5. The Expression of Tight Junction Genes and MUC2 Protein After CLP Surgery in Gut Microbiota-Transferred Mice. (A) Sections were prepared from the ileum of none-treat *WT* and *Gpr109a*$^{-/-}$ mice, and microbiota-transferred *WT* and *Gpr109a*$^{-/-}$ mice which undergo the CLP surgery (n = 3). The nucleus stained with DAPI is shown in blue. MUC2 is shown in green. The sections were observed by fluorescence microscopy. Magnification shown is 10×. The scale bar represents 500 µm. (B-G) Representative expression levels of *Cldn1*, *Cldn2*, *Cldn3*, *Zo1*, *Zo2*, and *Ocln* in ileum tissue homogenates from none-treat *WT* and *Gpr109a*$^{-/-}$ mice, and microbiota-transferred *WT* and *Gpr109a*$^{-/-}$ mice which undergo the CLP surgery (n = 3, three determinations per mouse). The results shown are means ± SEM, *$p < 0.05$.
